# Screen time and sweet consumption in preschool children: a cross-sectional study

**DOI:** 10.1590/0034-7167-2025-0461

**Published:** 2026-07-31

**Authors:** Soraya Alves Marreiro, Isabela Araújo Linhares Castro, Samir Gabriel Vasconcelos Azevedo, Thais Rodrigues de Albuquerque, Yenny Constanza Torres Rojas, Thaís Aquino Carneiro, Mariana Cavalcante Martins, Fabiane do Amaral Gubert

**Affiliations:** I Universidade Federal do Ceará. Fortaleza, Ceará, Brazil

**Keywords:** Screen Time, Child Nutrition, Preschool, Cross-Sectional Studies, Nursing., Tiempo de Pantalla, Nutrición del Niño, Preescolar, Estudios Transversales, Enfermería.

## Abstract

**Objectives:**

to analyze television and smartphone screen time and sweet consumption among preschool children in a capital city in northeastern Brazil.

**Methods:**

a cross-sectional study was conducted with 175 caregivers of children aged 1 to 5 years enrolled in six Early Childhood Education Centers. Sociodemographic data, screen time, and frequency of consumption of sweets, snacks, and soft drinks were collected and analyzed using non-parametric tests (p<0.05).

**Results:**

excessive smartphone use (>two hours/day) was significantly more frequent in children aged 3 to 4 years (Odds Ratio=2.26; 95% Confidence Interval: 1.09-4.67; p=0.02), of mixed race/color (p<0.01), and not enrolled in full-time education (p<0.01). Limits on candy consumption reduced the chance of excessive smartphone screen time (Odds Ratio=0.32; p=0.023).

**Conclusions:**

preschool children who exceed two hours of screen time per day showed higher intake of sweets.

## RESUMEN

**Objetivos:**

analizar el tiempo de uso de la televisión y los smartphones, así como el consumo de dulces en niños en edad preescolar de una capital del noreste de Brasil.

**Métodos:**

estudio transversal con 175 cuidadores de niños de 1 a 5 años matriculados en seis Centros de Educación Infantil. Se recopilaron datos sociodemográficos, el tiempo de uso de la televisión y la frecuencia de consumo de dulces, snacks y refrescos, y se analizaron mediante pruebas no paramétricas (p<0,05).

**Resultados:**

el uso excesivo de smartphones (>dos horas/día) fue significativamente más frecuente en niños de 3 a 4 años (*Odds Ratio*=2,26; Intervalo de Confianza del 95 %: 1,09-4,67; p=0,02), de raza mixta/color (p<0,01) y no matriculados en la escuela de tiempo completo (p<0,01). Limitar el consumo de dulces redujo la probabilidad de pasar demasiado tiempo frente a la pantalla del teléfono inteligente (*Odds Ratio*=0,32; p=0,023).

**Conclusiones:**

los niños en edad preescolar que superan las dos horas de tiempo frente a la pantalla al día mostraron un mayor consumo de dulces.

## INTRODUCTION

Screen time refers to the duration spent on entertainment activities mediated by electronic devices, such as televisions, smartphones, computers, and other technologies with a visual interface, generally characterized by passive content consumption. The World Health Organization (WHO) recommends that children under 2 years of age do not use screens and that those up to 5 years of age do not exceed one hour per day^(1-3)^. However, a meta-analysis revealed that 24.5% of children under 2 years old already use screens, and in the preschool phase this number can reach 35.6%, indicating excessive use^(4)^.

Prolonged screen time has been linked to several negative repercussions on children’s health, with effects that can extend into adolescence and adulthood. Evidence points to delays in cognitive, social, and linguistic skills^(5)^; poorer academic performance; reduced quality of interaction between parents and children^(6)^; and an increase in psychological and emotional problems, such as symptoms of depression, anxiety, and manifestations consistent with neurodevelopmental disorders. Additionally, there is a worsening of sleep quality^(7,8)^, an increase in disruptive behaviors^(7)^, as well as a higher risk of childhood obesity due to reduced physical activity and the prevalence of sedentary lifestyles^(5,9)^.

Furthermore, screen time directs children to advertising for unhealthy foods and influences their consumption patterns^(10)^, as observed by the relationship between excessive screen time and rejection of fruits and vegetables by Chinese preschool children^(11)^. In Spain, longer periods of screen time for children during their leisure time have been associated with poorer eating habits^(12)^. This reality is also present in the Brazilian context, where a relationship was found between screen time and the practice of eating meals in front of screens, as well as the consumption of ultra-processed foods by 4-year-old children^(13)^.

Despite existing evidence linking excessive screen time and sugar consumption in preschool children, it is necessary to understand how parents and guardians regulate, or fail to regulate, the use of electronic devices and access to ultra-processed foods in children’s daily lives. Therefore, investigating parental practices is fundamental to guiding more effective interventions by healthcare professionals, such as primary care nurses, who work directly with families and can promote more protective routines for children’s health^(11,14,15)^.

Studies investigating the effectiveness of nurse-led interventions to reduce screen time and sweet consumption by preschoolers have yielded results below expectations, such as the limited effectiveness of short-term interventions (sustainability of the effect)^(16)^ or even the inability to produce the primary effect, as exemplified by the responsive parenting intervention INSIGHT, which reduced screen time and television exposure in infants, but did not increase the frequency or amount of interactive play^(17)^.

Nurses play a central role in providing nutritional counseling to caregivers during early childhood, promoting healthy eating by adapting their guidance to the individual needs of each family. However, they face challenges in seeking up-to-date knowledge about nutrition and encounter difficulties when addressing sensitive topics such as childhood obesity^(18)^.

Thus, when considering the potential negative outcomes of inadequate nutrition in childhood, it becomes relevant to explore factors associated with food consumption. The aim is to produce scientific knowledge to support more precise health promotion strategies, foster public policies^(19)^ and guide the role of nursing during well-child visits and school health consultations, under a broader approach^(1)^.

## OBJECTIVES

To analyze television and smartphone screen time and sweet consumption among preschool children in a capital city in northeastern Brazil.

## METHODS

### Ethical aspects

In compliance with Resolution 466/12 of the Brazilian National Health Council, this study was approved by the Research Ethics Committee of the authors’ home institution, which issued an approval opinion. The Informed Consent Form was signed voluntarily by all participants.

### Study design, period and location

This is a cross-sectional study with a quantitative approach, guided by the STrengthening the Reporting of OBservational studies in Epidemiology checklist. It is a subset of the baseline of a cluster-randomized multicenter trial conducted in six public Early Childhood Education Centers (ECECs) in Fortaleza (Ceará), which serve children from 6 months to 5 years old.

ECECs, formerly known as daycare centers, are institutions specializing in the care and education of children from six months of age, through active pedagogical practices focused on holistic development in early childhood. These units offer full-time and part-time care options, organized in morning and afternoon shifts, according to demand and local organization.

At the time of data collection, the capital city had 186 public ECECs, serving approximately 25.556 children. For this study, the Municipal Department of Education indicated units located in areas of greater social vulnerability, based on the Social Vulnerability Index of *Instituto de Pesquisa Econômica Aplicada* ^(20,21)^. From this list, six ECECs were selected by simple random sampling, considering the age ranges of early childhood education I to IV (from 1 to 5 years old). Data collection took place between December 2022 and July 2023^(22)^.

### Population and inclusion criteria

Non-probabilistic convenience sampling was used, consisting of 175 guardians, defined as legal representatives or primary caregivers of children aged 1 to 5 years enrolled in childhood education I to IV classes of six ECECs. The sample size was derived from a larger longitudinal study, estimated based on the detection of a 0.2 kg/m^2^ difference in Body Mass Index (mean 19 kg/m^2^; Standard Deviation=0.05; α=0.05; power=90%; two-tailed), with an additional 20% to compensate for losses, totaling 99 participants per group. For this cross-sectional study, an average of 29 to 30 dyads per ECEC was considered. Guardians over 18 years of age, responsible for children aged 1 to 5 years, with active participation in daily care, were included. Guardians who did not respond after three contact attempts at different times were excluded. Although the Health Sciences Descriptors define preschoolers as children aged 2 to 5 years, and the international literature defines them as 3 to 5 years, this study adopted the 1-to-5-year age range, in accordance with the Brazilian educational classification.

### Study protocol

The questionnaire involved children’s sociodemographic variables (age, sex, race/color, school shift) and family composition (age and education of main caregivers), based on the economic classification criteria of *Associação Brasileira de Empresas de Pesquisa* (ABEP)^(23)^. Education was self-reported by the tutor and categorized into primary, secondary and higher education (complete or incomplete). Socioeconomic stratum was classified into groups A-B and C-D-E, according to the ABEP methodology.

Dependent variables were screen time and sweet consumption, investigated using instruments previously validated for the Brazilian context. To assess screen time, the Family Health Behavior Questionnaire^(24)^ was used, in which caregivers reported, in hours and minutes, the mean daily time children spent performing different activities, including playing outdoors, watching television/DVD, using a computer and/or smartphone and/or tablet, and playing video games, in addition to sleep times related to sleeping and waking. Estimates were collected separately for weekdays and weekends.

Based on WHO guidelines for children under 5 years old, the ideal parameters considered were: screen time ≤ 60 minutes/day, physical activity ≥ 180 minutes/day, and at least 11 hours of sleep for 2-year-olds and ten hours for those between 3 and 5 years old^(2)^.

Sweet consumption was assessed using the Parent Mealtime Action Scale^(25)^, which investigates how often guardians set limits on the daily consumption of sweets, soft drinks, and snacks, in three categories (1 - never, 2 - sometimes, 3 - always). An item from the Ministry of Health’s Food Consumption Markers was also included, asking whether or not meals occur in the presence of screens (answers: “yes”, “no” or “do not know”)^(26)^.

Tutors were initially contacted by phone or messaging app to schedule the interviews. Data collection was conducted in person, in private rooms at the ECECs, with only the researcher and the participant present. The researchers were previously trained on completing the instruments, standardizing the approaches, and conducting data collection ethically.

### Analysis of results and statistics

Data were analyzed using the Statistical Package for the Social Sciences version 27 after being entered into Microsoft Excel^®^ 2019. Due to the non-normality of continuous variables (p<0.05), non-parametric tests such as Mann-Whitney, Kruskal-Wallis, and Spearman correlation were used. Associations among categorical variables were verified using the chi-square test and, when necessary, Fisher’s exact test. To measure the strength of these associations, the unadjusted Odds Ratio (OR) was calculated, accompanied by a 95% Confidence Interval (95%CI). Cramer’s V coefficient was also calculated when pertinent. A statistical significance level of p<0.05 was adopted in all analyses. Omitted cases were excluded from the analysis and not replaced.

## RESULTS

The study included 175 caregivers, predominantly mothers (87.4%). Most families belonged to the C-D-E socioeconomic stratum (90.9%); and 73.7% were beneficiaries of the *Bolsa Família* Program (Family Allowance). Among children, 59.1% were between 1 and 2 years old; 71.5% were non-white (predominantly mixed race); and 81.5% were enrolled full-time in early childhood education centers. Most mothers had up to 8 years of education (92.6%). Table 1 presents participant sociodemographic characteristics.

**Table 1:** Research participant sociodemographic characteristics, Fortaleza, Ceará, Brazil, 2023

Variáveis (n: 175)		n	%
Children’s age in years (n=171)	1 - 2	101	59.1%
	3 - 4	70	40.9%
Sex (n=174)	Female	86	49.4%
	Male	88	50.6%
Skin color declared by parents/information about children (n=172)	White	49	28.5%
	Non-white	123	71.5%
Beneficiary of the Bolsa Família Program (n=171)	Yes	126	73.7%
	No	45	26.3%
Period the child attends daycare (n=173)	Full-time	141	81.5%
	Non-full-time	32	18.5%
Relationship of the child (n=174)	Mother	152	87.4%
	Other caregiver	22	12.6%
Mother’s education level (n=95)	> eight years of education	7	7.4%
	Up to eight years of education	88	92.6%
Socioeconomic status (n=174)	A-B	16	9.2%
	C-D-E	158	90.9%

*Legend: the variation in the number of responses (n) among variables is due to some participants not responding to certain items on the questionnaire.*

The median daily screen time (television and smartphone) was 60 minutes on weekdays, increasing on weekends, when more than 50% of children exceeded two hours per day. Excessive smartphone use (>two hours/day) was significantly more frequent in children aged 3 to 4 years (OR=2.26; 95%CI: 1.09-4.67; p=0.02), of mixed race/color (p<0.01), and among those not enrolled in full-time schooling (p<0.01). For television, there was no significant difference by age. Only a trend towards a higher proportion of >two hours/day was observed among children aged 3-4 years (p=0.11 on weekends; p=0.57 during the week).

There was a significant association between race/skin color and screen time, with children of mixed race/color showing greater exposure to both TV (p=0.02) and smartphones (p<0.01). Table 2 presents detailed data on screen time according to age and race/color. Full-time attendance at ECCs was associated with less smartphone use during the week and weekends (p<0.01), indicating a possible protective effect of structured school routines.

**Table 2:** Screen time by age, Fortaleza, Ceará, Brazil, 2023

Screen time	Age									*p*
	1-2 years				3-4 years					
	n	%	95%CI		n	%	95%CI		OR [95%CI]	
**Television during the week**									-	0.57
≤ two hours	76	75.2	66.2	82.9	50	71.4	60.1	81.0		
> two hours	25	24.8	17.1	33.8	20	28.6	19.0	39.9		
**Television on the weekend**									-	0.11
≤ two hours	71	70.3	60.9	78.5	41	58.6	46.9	69.6		
> two hours	30	29.7	21.5	39.1	29	41.4	30.4	53.1		
***Smartphone during the week***									2.26 [1.09-4.67]	0.02^*^
≤ two hours	84	83.2	75.0	89.5	48	68.6	57.1	78.5		
> two hours	17	16.8	10.5	25.0	22	31.4	21.5	42.9		
***Smartphone on the weekend***									2.39 [1.20-4.76]	0.01^*^
≤ two hours	81	80.2	71.6	87.1	44	62.9	51.2	73.5		
> two hours	20	19.8	12.9	28.4	26	37.1	26.5	48.8		

* *Chi-square test; OR - Odds Ratio.*

Concerning eating behavior, more than half of children (59.8%) ate meals in front of screens, a practice more common among children aged 1 to 2 years (63.9%). Sweet consumption during meals in front of screens was higher in this group. About 28% of children received additional screen time as a reward from their caregivers.

Although 66% of tutors reported imposing limits on screen time, such restrictions were not significantly associated with a reduction in screen time, suggesting low effectiveness of parental strategies (Table 3).

**Table 3:** Screen time (television and smartphone) during the week and on weekends according to screen time limits reported by children’s guardians, Fortaleza, Ceará, Brazil, 2023 (N=175)

Screen time	Daily time	Limit imposed by tutors	n	%	*p* value
**Television (week)**	≤ two hours	Yes	102	75.6	0.18
		No	26	65.0	
	> two hours	Yes	33	24.4	
		No	14	35.0	
**Smartphone (week)**	≤ two hours	Yes	105	77.8	0.71
		No	30	75.0	
	> two hours	Yes	30	22.2	
		No	10	25.0	
**Television (weekend)**	≤ two hours	Yes	81	65.9	0.95
		No	34	65.4	
	> two hours	Yes	42	34.1	
		No	18	34.6	
**Smartphone (weekend)**	≤ two hours	Yes	93	75.6	0.25
		No	35	67.3	
	> two hours	Yes	30	24.4	
		No	17	32.7	

*Legend: the “n” values per category refer only to participants who responded to the respective variable. Screen time categories are mutually exclusive (≤ two hours or > two hours per day). The variation in totals is due to missing responses (data omitted) in some questionnaire items.*

Limits on sweet consumption were associated with a lower chance of excessive screen time only for smartphones (OR=0.32; 95%CI: 0.12-0.85; p=0.023). For television, the association was not significant (OR=1.63; p=0.445). For soft drinks and snacks, we did not observe significant associations with >two hours/day of screen time (TV or smartphone), as shown in Table 4.

**Table 4:** Excessive screen time (television and smartphone) related to limits on sweet consumption, Fortaleza, Ceará, Brazil, 2023 (N=175)

Type of treat	Device	Limit imposed by tutors	>two hours of screen time (n. %)	OR	*p* value
**Candy**	TV	Yes	42 (36.8%)	1.63	0.445
		No	5 (26.3%)	Ref.	-
	Smartphone	Yes	28 (27.7%)	0.32	0.023^*^
		No	12 (54.5%)	Ref.	-
**Soft drinks**	TV	Yes	32 (34.8%)	0.71	0.417
		No	15 (42.9%)	Ref.	-
	Smartphone	Yes	28 (29.2%)	0.48	0.156
		No	12 (46.2%)	Ref.	-
**Snacks**	TV	Yes	38 (37.6%)	1.21	0.823
		No	9 (33.3%)	Ref.	-
	Smartphone	Yes	30 (31.3%)	0.64	0.343
		No	10 (41.7%)	Ref.	-

*Legend: the Odds Ratio was calculated by comparing “enforces limit” versus “does not enforce” (reference); unadjusted ORs; p-values by Fisher’s exact test (when applicable) or chi-square; significance level: p<0.05.*

These results indicate that parental dietary control exercised through limits on sweet consumption was associated with a lower likelihood of excessive smartphone use, but this did not extend to television nor was it consistent for other types of ultra-processed foods.

## DISCUSSION

This study revealed that children, especially those aged 3 to 4, of mixed race/color, and not enrolled in full-time education, showed a higher frequency of excessive smartphone use. Regarding screen time, it was found that more than half of children ate meals in front of screens, a behavior associated with increased sweet consumption.

The results of this study reveal a worrying picture of the early and excessive use of electronic devices among preschool children, especially smartphones, whose frequency of use was significantly higher among children aged 3 to 4 years. This finding is in line with guidelines from the WHO^(2)^, the American Academy of Pediatrics^(25)^ and the Brazilian Society of Pediatrics^(26)^, which recommend completely avoiding screen use in children under 2 years of age and limiting it to a maximum of one hour per day between two and 5 years of age. The high proportion of children aged 1 to 2 years who already exceeded the recommended screen time limits stands out as a worrying finding.

The association between older age and greater screen exposure may reflect both increased childhood autonomy and reduced parental supervision as a child grows, a factor identified in the literature as a risk factor for the development of harmful digital behaviors^(5,6)^.

Another relevant finding was that more than half of children ate meals in front of screens, mostly those aged 1 to 2 years. This behavior compromises the self-regulation of hunger and satiety, favoring sweet consumption and disorganized eating habits^(23)^. The literature also points to risks of selective eating, food refusal, and excessive calorie intake when eating occurs in front of devices^(27,28)^. The high prevalence of children between one and 2 years old consuming sweets and excessive screen time may be associated with the period of food introduction, since caregivers use screens to distract the child and thus feed them, a habit that can persist over time.

In relation to sweet consumption, most caregivers reported setting limits, especially for sweets (66.3%). It was observed that such limits were associated with a lower chance of excessive screen time when using smartphones. However, for television and the other ultra-processed foods assessed (soft drinks and snacks), the associations were not statistically significant. This finding suggests that imposing limits on screen time alone proved to be ineffective in reducing exposure, a fact also reported in other contexts^(29)^, indicating that interventions focusing on education and parental supervision are necessary for the effective reduction of exposure time. Evidence suggests that multifaceted strategies, such as parental education^(30)^, the use of digital technologies for disseminating information^(23)^, and the provision of alternative activities^(31,32)^ are more effective in reducing screen time in preschoolers. Incorporating these strategies into primary health care programs and the routine of daycare centers can contribute to the promotion of children’s health and well-being.

The weekend emerged as a critical moment, with increased screen time and higher consumption of soft drinks. This relaxation of routines, coupled with the distraction caused by devices, can encourage the consumption of ultra-processed foods, especially sugary drinks^(33)^. Furthermore, parental eating habits can influence this intake, causing children to consume more soda compared to other foods, such as fruits and vegetables^(13)^.

The association between increased screen time and mixed race/color highlights structural inequalities that impact children’s digital and dietary habits. Studies suggest that black and mixed race children are more likely to be exposed to modifiable risk factors, such as prolonged screen time and inadequate nutrition, reflecting social vulnerability and less access to protective environments^(34,35)^. The predominance of children who identified as mixed-race in this study highlights the importance of considering racial factors in analyses of parenting practices, screen time, and eating behaviors in early childhood.

The amount of time a child spends at school directly influences their screen time (television and smartphone use). It was observed that children enrolled in full-time programs at ECECs showed less screen time, reinforcing the protective role of the school routine, as described in other studies^(36,37)^. Another study adds comparisons between different ages, showing that younger children accumulate more weekly screen hours than older children, suggesting that entering and remaining in ECECs contributes to gradually reducing this exposure^(38)^.

The high proportion of families benefiting from income transfer programs highlights the influence of socioeconomic vulnerability on children’s screen time. Although children from wealthier families have greater access to devices, research indicates that in vulnerable contexts, screen use is even more frequent^(39)^. In these contexts, screens are frequently used as the primary form of leisure, given the scarcity of alternatives, which can exacerbate inadequate eating habits, as demonstrated by national and international studies^(40,41)^.

Another relevant finding was the high proportion of mothers with up to eight years of education, reflecting a scenario of low maternal education. This factor, associated with the unfavorable socioeconomic context, can influence both children’s screen time and dietary patterns, as already described in national and international studies that link lower parental education to less protective health practices^(18,34,35)^.

The relationship between screen time and setting limits was also explored. Most caregivers reported imposing restrictions on device use, with a higher rate among children who spend up to two hours a day in front of screens. However, no statistically significant association was identified between setting these limits and reducing screen time, suggesting that, even with a declared intention to control, many caregivers are unable to effectively moderate children’s screen use. This result points to a possible gap between parental practice and children’s actual behavior, a finding also observed in a Canadian study that compared monitoring and limiting screen time during the week with actual screen time, with parental limitations not directly reflected in less screen time^(42)^.

Given this, sweet consumption in front of screens, even with limits set by guardians, is a worrying finding. The intake of sweets associated with excessive screen time can contribute to unregulated eating patterns and greater vulnerability to health problems throughout life^(43)^. Viola *et al*.^(18)^ found a correlation between increased screen time and higher intake of ultra-processed foods, such as sweets and sugary drinks, suggesting that prolonged time on devices may act as a facilitating factor for the adoption of unhealthy eating habits. Similarly, Mumena *et al*.^(44)^ highlight that excessive screen use, especially during leisure time, is associated with increased consumption of sugary foods, supporting the idea that excessive screen time not only affects eating behavior but also the development of long-term eating habits.

In this context, the use of screens and sweet consumption are proving to be a risky practice, increasingly incorporated into families, which harms not only the child physically, but also the behavioral, emotional, and social characteristics developed throughout childhood.

### Study limitations

Since this is a cross-sectional study, it is not possible to establish causal relationships between screen time, sociodemographic variables, and sweet consumption. The observed associations should therefore be interpreted as correlational. Longitudinal studies are recommended to better understand the effects of screen time on dietary patterns throughout childhood development. Furthermore, the sample consisted of children enrolled in public early childhood education centers in a northeastern municipality, which may limit the generalizability of the findings to other populations with different socioeconomic and cultural profiles. Additionally, screen time was estimated through self-reporting by caregivers, which may have introduced recall bias or social desirability bias. Finally, qualitative aspects of the content and the context of screen use, relevant elements for interpreting their impacts on development, were not assessed.

### Contributions to nursing, health, or public policy

The findings of this study offer relevant insights for nurses working in Primary Health Care and ECEC units, with the goal of planning preventive actions focused on preschool children’s digital and nutritional health. Hence, nurses can incorporate into their care routine the assessment of screen time and eating habits during well-child visits, using brief screening tools and creating protocols to identify risk situations. The nurses’ role can include developing educational workshops with parents and caregivers, discussion groups on appropriate screen time management, and the creation of educational materials on healthy eating in early childhood. Nurses can also work in an intersectoral manner, integrating nutritional surveillance and parental guidance actions, strengthening public policies focused on the comprehensive health of children.

## CONCLUSIONS

The study showed that preschool children who exceed two hours of screen time per day had higher intake of soft drinks and sweets, especially when eating in front of screens, with a higher occurrence on weekends, suggesting less parental control. Children in full-time daycare had less screen time, indicating the protective role of structured environments and routines.

Structural factors, such as low maternal education, unfavorable socioeconomic status, and self-declared mixed race, emerged as critical determinants of greater screen exposure and inadequate dietary patterns. These results reinforce the need for intersectoral interventions, with an emphasis on the role of nursing in the context of Primary Health Care, aimed at promoting healthy screen use habits and limiting screen time during meals, in order to reduce sweet consumption by preschoolers.

## Data Availability

Research data may be made available upon request to the corresponding author.
