# Supplementary material for: Screen time and sweet consumption in preschool children: a cross-sectional study
Source: Rev Bras Enferm. 2026 Jul 31;79:e20250461. doi: 10.1590/0034-7167-2025-0461 (PMC13435861; doi:10.1590/0034-7167-2025-0461)
Supplement: Translation

# Translation: Tempo de tela e consumo de guloseimas em crianças pré-escolares: estudo transversal

**DOI:** 10.1590/0034-7167-2025-0461pt

**Authors:** Soraya Alves Marreiro, Isabela Araújo Linhares Castro, Samir Gabriel Vasconcelos Azevedo, Thais Rodrigues de Albuquerque, Yenny Constanza Torres Rojas, Thaís Aquino Carneiro, Mariana Cavalcante Martins, Fabiane do Amaral Gubert

**Affiliations:** I Universidade Federal do Ceará. Fortaleza, Ceará, Brasil

**Keywords:** Tempo de Tela, Nutrição da Criança, Crianças Pré-Escolares, Estudos Transversais, Enfermagem.

## Abstract

**Objetivo:**

analisar o tempo de exposição à televisão e *smartphone* e o consumo de guloseimas entre as crianças pré-escolares em uma capital do nordeste brasileiro.

**Métodos:**

estudo transversal com 175 tutores de crianças de 1 a 5 anos matriculadas em seis Centros de Educação Infantil. Coletaram-se dados sociodemográficos, tempo de tela, e frequência de consumo de doces, salgadinhos e refrigerantes, analisados por testes não paramétricos (p<0,05).

**Resultados:**

o uso excessivo de *smartphone* (>duas horas/dia) foi significativamente mais frequente em crianças de 3 a 4 anos (*Odds Ratio*=2,26; Intervalo de Confiança de 95%: 1,09-4,67; p=0,02), de raça/cor parda (p<0,01) e não matriculadas em período integral (p<0,01). Limites ao consumo de doces reduziram a chance de tempo de tela excessivo no *smartphone* (*Odds Ratio*=0,32; p=0,023).

**Conclusões:**

crianças pré-escolares que excedem duas horas diárias de tela apresentaram maior ingestão de guloseimas.

## INTRODUÇÃO

O tempo de tela corresponde à duração dedicada a atividades de entretenimento mediadas por dispositivos eletrônicos, como televisores, *smartphones*, computadores e demais tecnologias com interface visual, geralmente caracterizadas por um consumo passivo de conteúdo. A Organização Mundial da Saúde (OMS) recomenda que crianças menores de 2 anos não utilizem telas e que aquelas de até 5 anos não ultrapassem uma hora diária^(1-3)^. No entanto, metanálise revelou que 24,5% das crianças menores de 2 anos já utilizam telas, e na fase pré-escolar, esse número pode chegar a 35,6%, indicando uso excessivo^(4)^.

A exposição prolongada às telas tem sido associada a diversas repercussões negativas na saúde infantil, com efeitos que podem se estender para a adolescência e vida adulta. Evidências apontam atrasos nas habilidades cognitivas, sociais e linguísticas^(5)^; pior desempenho acadêmico; redução da qualidade de interação entre pais e filhos^(6)^; e aumento de problemas psicológicos e emocionais, como sintomas de depressão, ansiedade e manifestações compatíveis com transtornos do neurodesenvolvimento. Adicionalmente, ocorrem piora na qualidade do sono^(7,8)^, aumento de comportamentos disruptivos^(7)^, bem como maior risco de obesidade infantil devido à redução da atividade física e prevalência do sedentarismo^(5,9)^.

Além disso, o tempo de tela direciona as crianças à publicidade de alimentos não saudáveis e influencia seus padrões de consumo^(10)^, como observado pela relação entre tempo excessivo em telas e rejeição de frutas e vegetais por crianças chinesas em idade pré-escolar^(11)^. Na Espanha, períodos mais longos de crianças em frente às telas durante seu tempo de lazer foram associados a comportamentos alimentares mais precários^(12)^. Essa realidade também se mostra presente no contexto brasileiro, na qual se encontrou uma relação entre a exposição às telas e a prática de fazer refeições diante das telas, bem como ao consumo de alimentos ultraprocessados por crianças de 4 anos^(13)^.

Apesar das evidências existentes sobre a associação entre tempo excessivo de tela e consumo de guloseimas em crianças pré-escolares, é necessário compreender como pais e tutores regulam ou deixam de regular o uso de dispositivos eletrônicos e o acesso a alimentos ultraprocessados no cotidiano infantil. Assim, investigar as práticas parentais é fundamental para orientar intervenções mais eficazes por profissionais de saúde, como enfermeiros da atenção primária, que atuam diretamente com famílias e podem promover rotinas mais protetivas à saúde infantil^(11,14,15)^.

Os estudos que investigaram a eficácia de intervenções conduzidas por enfermeiros para reduzir o tempo de tela e o consumo alimentar de guloseimas por pré-escolares apresentaram resultados aquém do esperado, como a eficácia limitada das intervenções de curta duração (sustentabilidade do efeito)^(16)^ ou mesmo a incapacidade de produzir o efeito primário, a exemplo da intervenção parental responsiva *INSIGHT*, que reduziu o tempo de tela e a exposição à televisão em bebês, mas não aumentou a frequência ou a quantidade de brincadeiras interativas^(17)^.

Enfermeiros desempenham um papel central no aconselhamento nutricional dos tutores durante a primeira infância, que conseguem promover alimentação saudável ao adaptarem suas orientações de acordo com as necessidades individuais de cada família. Contudo, enfrentam desafios na busca por conhecimento atualizado sobre nutrição e encontram dificuldades ao abordar temas sensíveis como obesidade infantil^(18)^.

Dessa maneira, ao considerar os potenciais desfechos negativos da alimentação inadequada na infância, torna-se relevante explorar os fatores associados ao consumo alimentar. Objetiva-se produzir conhecimento científico para fundamentar estratégias mais precisas de promoção da saúde, fomentar políticas públicas^(19)^ e direcionar o papel da enfermagem durante as consultas de puericultura e de saúde escolar, sob uma abordagem ampliada^(1)^.

## OBJETIVOS

Analisar o tempo de exposição à televisão e *smartphone* e o consumo de guloseimas entre as crianças pré-escolares em uma capital do nordeste brasileiro.

## MÉTODOS

### Aspectos éticos

Em cumprimento à Resolução nº 466/12 do Conselho Nacional de Saúde, este estudo foi aprovado pelo Comitê de Ética em Pesquisa da instituição de origem dos autores, que emitiu parecer aprovado. O Termo de Consentimento Livre e Esclarecido foi assinado espontaneamente por todos os participantes.

### Desenho, período e local do estudo

Trata-se de estudo transversal, com abordagem quantitativa, norteado pela lista de verificação *STrengthening the Reporting of OBservacional Studies in Epidemiology*. Trata-se de recorte da linha de base de ensaio multicêntrico randomizado por *clusters*, desenvolvido em seis Centros de Educação Infantil (CEIs) públicos de Fortaleza (Ceará), que atendem crianças de 6 meses a 5 anos.

Os CEIs, anteriormente conhecidos como creches, são instituições especializadas na promoção do cuidado e da educação de crianças a partir dos 6 meses de vida, por meio de práticas pedagógicas ativas voltadas ao desenvolvimento integral na primeira infância. Essas unidades ofertam modalidades de atendimento em tempo integral e parcial, organizadas em turnos matutinos e vespertinos, conforme a demanda e a organização local.

À época da coleta, a capital contava com 186 CEIs públicos, atendendo aproximadamente 25.556 crianças. Para este estudo, a Secretaria Municipal de Educação indicou unidades localizadas em áreas de maior vulnerabilidade social, com base no Índice de Vulnerabilidade Social do Instituto de Pesquisa Econômica Aplicada^(20,21)^. A partir dessa lista, seis CEIs foram selecionados por sorteio aleatório simples, considerando as faixas etárias do ensino infantil I ao IV (de 1 a 5 anos). A coleta de dados ocorreu entre dezembro de 2022 e julho de 2023^(22)^.

### População e critérios de inclusão

Utilizou-se amostragem não probabilística por conveniência, composta por 175 tutores, definidos como responsáveis legais ou cuidadores principais de crianças de 1 a 5 anos matriculadas nas turmas infantil I a IV de seis CEIs. O tamanho amostral foi derivado de pesquisa longitudinal maior, estimado com base na detecção de uma diferença de 0,2 kg/m^2^ no Índice de Massa Corporal (média de 19 kg/m^2^; Desvio Padrão=0,05; α=0,05; poder=90%; bicaudal), com acréscimo de 20% para compensar perdas, totalizando 99 participantes por grupo. Para este recorte transversal, considerou-se uma média de 29 a 30 díades por CEI. Foram incluídos tutores com mais de 18 anos, responsáveis por crianças de 1 a 5 anos, com participação ativa nos cuidados diários. Foram excluídos os que não responderam após três tentativas de contato em horários distintos. Embora os Descritores em Ciências da Saúde definam pré-escolares como crianças de 2 a 5 anos, e a literatura internacional, de 3 a 5, neste estudo, adotou-se a faixa 1 a 5 anos, em consonância com a classificação educacional brasileira.

### Protocolo do estudo

O questionário envolveu as variáveis sociodemográficas das crianças (idade, sexo, raça/cor, turno escolar) e da composição familiar (idade e escolaridade do cuidador principal), baseado no critério de classificação econômica da Associação Brasileira de Empresas de Pesquisa (ABEP)^(23)^. A escolaridade foi autorreferida pelo tutor e categorizada em ensino fundamental, médio e superior (completo ou incompleto). O estrato socioeconômico foi classificado nos grupos A-B e C-D-E, conforme a metodologia da ABEP.

As variáveis dependentes foram o tempo de exposição às telas e o consumo de guloseimas, investigadas com o uso de instrumentos validados previamente para o contexto brasileiro. Para avaliação do tempo de tela, foi utilizado o Questionário de Comportamentos em Saúde da Família^(24)^, no qual os tutores relataram, em horas e minutos, o tempo médio diário que a criança passava realizando diferentes atividades, incluindo brincar ao ar livre, assistir televisão/DVD, usar computador e/ou *smartphone* e/ou *tablet* e jogar *videogame*, além dos horários de sono relacionados ao dormir e acordar. As estimativas foram coletadas separadamente para dias de semana e finais de semana.

Com base nas diretrizes da OMS para crianças menores de 5 anos, os parâmetros considerados ideais foram tempo de tela ≤ 60 minutos/dia, atividade física ≥ 180 minutos/dia e sono de pelo menos 11 horas, para crianças de 2 anos, e dez horas, para aquelas entre 3 e 5 anos^(2)^.

O consumo de guloseimas foi avaliado por meio da Escala de Comportamento dos Pais Durante as Refeições^(25)^, que investiga a frequência com que os tutores impõem limites ao consumo diário de doces, refrigerantes e salgadinhos, em três categorias (1 - nunca, 2 - às vezes, 3 - sempre). Também foi incluído um item dos Marcadores de Consumo Alimentar do Ministério da Saúde, questionando se as refeições ocorrem ou não na presença de telas (respostas: “sim”, “não” ou “não sabe”)^(26)^.

Os tutores foram inicialmente contatados por telefone ou aplicativo de mensagens para agendamento das entrevistas. A coleta de dados foi realizada presencialmente, em salas privativas nos CEIs, com a presença exclusiva do pesquisador e do participante. Os pesquisadores foram previamente treinados quanto ao preenchimento dos instrumentos, padronização das abordagens e condução ética da coleta.

### Análise dos resultados e estatística

Os dados foram analisados no *Statistical Package for the Social Sciences*, versão 27, após digitação no *Microsoft Excel* ^®^ 2019. Devido à não normalidade das variáveis contínuas (p<0,05), utilizaram-se testes não paramétricos como Mann-Whitney, Kruskal-Wallis e correlação de Spearman. Associações entre variáveis categóricas foram verificadas por teste qui-quadrado e, quando necessário, teste exato de Fisher. Para mensurar a força dessas associações, calculou-se o *Odds Ratio* (OR) bruto, acompanhado de Intervalo de Confiança de 95% (IC95%). Também foi calculado o coeficiente V de Cramer quando pertinente. Adotou-se nível de significância estatística de p<0,05 em todas as análises. Casos omissos foram excluídos da análise e não substituídos.

## RESULTADOS

Participaram do estudo 175 tutores, predominantemente mães (87,4%). A maioria das famílias pertencia ao estrato socioeconômico C-D-E (90,9%), e 73,7% eram beneficiárias do Programa Bolsa Família. Entre as crianças, 59,1% tinham entre 1 e 2 anos; 71,5% eram não brancas (predominantemente pardas); e 81,5% estavam matriculadas em regime integral nos CEIs. A maior parte das mães tinha até oito anos de estudo (92,6%). As características sociodemográficas dos participantes estão apresentadas na Tabela 1.

**Tabela 1:** Características sociodemográficas dos participantes da pesquisa, Fortaleza, Ceará, Brasil, 2023

Variáveis (n: 175)		n	%
Idade das crianças em anos (n=171)	1 - 2	101	59,1%
	3 - 4	70	40,9%
Sexo (n=174)	Feminino	86	49,4%
	Masculino	88	50,6%
Cor da pele declarada pelos pais/informação das crianças (n=172)	Branca	49	28,5%
	Não branca	123	71,5%
Beneficiária do Programa Bolsa Família (n=171)	Sim	126	73,7%
	Não	45	26,3%
Período que a criança frequenta a creche (n=173)	Integral	141	81,5%
	Não integral	32	18,5%
Parentesco da criança (n=174)	Mãe	152	87,4%
	Outro cuidador	22	12,6%
Escolaridade da mãe (n=95)	> oito anos de estudo	7	7,4%
	Até oito anos de estudo	88	92,6%
Estrato socioeconômico (n=174)	A-B	16	9,2%
	C-D-E	158	90,9%

*Legenda: a variação no número de respostas (n) entre as variáveis ocorreu devido à ausência de resposta por parte de alguns participantes em determinados itens do questionário.*

O tempo mediano de exposição diária às telas (televisão e *smartphone*) foi de 60 minutos nos dias de semana, com aumento nos fins de semana, quando mais de 50% das crianças ultrapassaram duas horas diárias. O uso excessivo de *smartphone* (>duas horas/dia) foi significativamente mais frequente em crianças de 3 a 4 anos (OR=2,26; IC95%: 1,09-4,67; p=0,02), de raça/cor parda (p<0,01) e entre aquelas não matriculadas em período integral (p<0,01). Para televisão, não houve diferença significativa por idade. Observou-se apenas tendência de maior proporção de >duas horas/dia entre crianças de 3-4 anos (p=0,11 no fim de semana; p=0,57 na semana).

Houve associação significativa entre a raça/cor da pele e o tempo de tela com crianças de raça/cor parda que apresentaram maior exposição tanto à TV (p=0,02) quanto ao *smartphone* (p<0,01). Os dados detalhados sobre tempo de tela segundo idade e raça/cor estão apresentados na Tabela 2. O regime integral nos CEIs se associou ao menor uso de *smartphones* durante a semana e fins de semana (p<0,01), indicando um possível efeito protetor das rotinas estruturadas escolares.

**Tabela 2:** Tempo de tela segundo idade, Fortaleza, Ceará, Brasil, 2023

Tempo de tela	Idade									*p*
	1-2 anos				3-4 anos					
	n	%	IC95%		n	%	IC95%		OR [IC95%]	
**Televisão na semana**									-	0,57
≤ duas horas	76	75,2	66,2	82,9	50	71,4	60,1	81,0		
> duas horas	25	24,8	17,1	33,8	20	28,6	19,0	39,9		
**Televisão no fim de semana**									-	0,11
≤ duas horas	71	70,3	60,9	78,5	41	58,6	46,9	69,6		
> duas horas	30	29,7	21,5	39,1	29	41,4	30,4	53,1		
***Smartphone* na semana**									2,26 [1,09-4,67]	0,02^*^
≤ duas horas	84	83,2	75,0	89,5	48	68,6	57,1	78,5		
> duas horas	17	16,8	10,5	25,0	22	31,4	21,5	42,9		
***Smartphone* no fim de semana**									2,39 [1,20-4,76]	0,01^*^
≤ duas horas	81	80,2	71,6	87,1	44	62,9	51,2	73,5		
> duas horas	20	19,8	12,9	28,4	26	37,1	26,5	48,8		

* *Teste qui-quadrado; OR - Odds Ratio.*

No tocante ao comportamento alimentar, mais da metade das crianças (59,8%) realizava refeições diante das telas, sendo essa prática mais comum entre crianças de 1 a 2 anos (63,9%). O consumo de guloseimas durante as refeições diante das telas foi mais elevado nesse grupo. Cerca de 28% das crianças recebiam tempo adicional de tela como recompensa dos tutores.

Embora 66% dos tutores referissem impor limites ao tempo de tela, tais restrições não se associaram significativamente à redução do tempo de exposição aos dispositivos, sugerindo baixa efetividade das estratégias parentais (Tabela 3).

**Tabela 3:** Tempo de tela (televisão e *smartphone*) na semana e finais de semana segundo limites de tempo de tela relatados pelos tutores das crianças, Fortaleza, Ceará, Brasil, 2023 (N=175)

Tempo de tela	Tempo diário	Limite imposto pelo tutor	n	%	Valor de *p*
**Televisão (semana)**	≤ duas horas	Sim	102	75,6	0,18
		Não	26	65,0	
	> duas horas	Sim	33	24,4	
		Não	14	35,0	
***Smartphone* (semana)**	≤ duas horas	Sim	105	77,8	0,71
		Não	30	75,0	
	> duas horas	Sim	30	22,2	
		Não	10	25,0	
**Televisão (fim de semana)**	≤ duas horas	Sim	81	65,9	0,95
		Não	34	65,4	
	> duas horas	Sim	42	34,1	
		Não	18	34,6	
***Smartphone* (fim de semana)**	≤ duas horas	Sim	93	75,6	0,25
		Não	35	67,3	
	> duas horas	Sim	30	24,4	
		Não	17	32,7	

*Legenda: os valores de “n” por categoria se referem apenas aos participantes que responderam à respectiva variável. As categorias de tempo de tela são mutuamente exclusivas (≤ duas horas ou > duas horas por dia). A variação nos totais deve-se à ausência de respostas (dados omitidos) em alguns itens do questionário.*

Limites ao consumo de doces se associaram a menor chance de tempo excessivo de tela apenas para *smartphone* (OR=0,32; IC95%: 0,12-0,85; p=0,023). Para televisão, a associação não foi significativa (OR=1,63; p=0,445). Para refrigerantes e salgadinhos, não observamos associações significativas com >duas horas/dia de tela (TV ou *smartphone*), conforme Tabela 4.

**Tabela 4:** Tempo de tela excessivo (televisão e *smartphone*) relacionado aos limites no consumo de guloseimas, Fortaleza, Ceará, Brasil, 2023 (N=175)

Tipo de guloseima	Dispositivo	Limite imposto pelo tutor	>duas horas de tela (n, %)	OR	Valor de *p*
**Doces**	TV	Sim	42 (36,8%)	1,63	0,445
		Não	5 (26,3%)	Ref.	-
	*Smartphone*	Sim	28 (27,7%)	0,32	0,023^*^
		Não	12 (54,5%)	Ref.	-
**Refrigerantes**	TV	Sim	32 (34,8%)	0,71	0,417
		Não	15 (42,9%)	Ref.	-
	*Smartphone*	Sim	28 (29,2%)	0,48	0,156
		Não	12 (46,2%)	Ref.	-
**Salgadinhos**	TV	Sim	38 (37,6%)	1,21	0,823
		Não	9 (33,3%)	Ref.	-
	*Smartphone*	Sim	30 (31,3%)	0,64	0,343
		Não	10 (41,7%)	Ref.	-

*Legenda: o Odds Ratio foi calculado comparando “impõe limite” versus “não impõe” (referência); OR brutos; valores de p por teste exato de Fisher (quando aplicável) ou qui-quadrado; nível de significância: p<0,05.*

Esses resultados indicam que o controle alimentar parental exercido por meio de limites ao consumo de doces se associou a menor chance de tempo excessivo no uso de *smartphone*, mas não se estendeu à televisão, nem se mostrou consistente para outros tipos de alimentos ultraprocessados.

## DISCUSSÃO

O presente estudo revelou que crianças, principalmente as de 3 a 4 anos, de raça/cor parda e não matriculadas em tempo integral, apresentaram maior frequência de uso excessivo de *smartphones*. Sobre o tempo de tela, verificou-se que mais da metade das crianças realizava refeições diante das telas, comportamento associado ao maior consumo de guloseimas.

Os resultados deste estudo revelam um panorama preocupante do uso precoce e excessivo de dispositivos eletrônicos entre crianças pré-escolares, principalmente o *smartphone*, cuja frequência de uso foi significativamente maior entre crianças de 3 a 4 anos. Essa constatação está em consonância com diretrizes da OMS^(2)^, da *American Academy of Pediatrics* ^(25)^ e da Sociedade Brasileira de Pediatria^(26)^, que recomendam evitar completamente o uso de telas em menores de 2 anos e limitá-lo a, no máximo, uma hora diária entre 2 e 5 anos. Destaca-se como achado preocupante a elevada proporção de crianças de 1 a 2 anos que já excediam os limites recomendados de tempo de tela.

A associação entre maior idade e maior exposição às telas pode refletir tanto o aumento da autonomia infantil quanto uma redução na supervisão parental conforme a criança cresce, fator identificado na literatura como de risco para o estabelecimento de comportamentos digitais nocivos^(5,6)^.

Outro achado relevante foi que mais da metade das crianças realizava refeições diante das telas, sendo a maioria as de 1 a 2 anos. Esse comportamento compromete a autorregulação da fome e da saciedade, favorecendo o consumo de guloseimas e hábitos alimentares desorganizados^(23)^. A literatura aponta ainda para riscos de seletividade, recusa alimentar e ingestão calórica excessiva quando a alimentação ocorre diante de dispositivos^(27,28)^. A alta prevalência de crianças entre 1 e 2 anos no consumo de guloseimas e excesso de telas pode estar associado ao período de introdução alimentar, uma vez que os tutores as utilizam na prerrogativa de distrair a criança e, assim, levar o alimento à boca, hábito esse que pode persistir com o passar do tempo.

Em relação ao consumo de guloseimas, a maioria dos cuidadores relatou impor limites, especialmente para doces (66,3%). Observou-se que tais limites se associaram a menor chance de tempo excessivo de tela no uso de *smartphone*. Porém, para televisão e os demais alimentos ultraprocessados avaliados (refrigerantes e salgadinhos), as associações não foram estatisticamente significativas. Esse achado sugere que a imposição de limites ao tempo de tela, unicamente, mostrou-se pouco efetiva para reduzir a exposição, fato também relatado em outros contextos^(29)^, indicando que intervenções com foco na educação e supervisão parental são necessárias para a redução efetiva do tempo de exposição. Evidências apontam que estratégias multifacetadas, como educação parental^(30)^, uso de tecnologias digitais para disseminação de informações^(23)^ e oferta de atividades alternativas^(31,32)^, são mais eficazes na redução do tempo de tela em pré-escolares. A incorporação dessas estratégias em programas de saúde primária e na rotina das creches pode contribuir para a promoção da saúde e o bem-estar infantil.

O fim de semana despontou como um momento crítico, com maior exposição às telas e maior consumo de refrigerantes. Essa flexibilização das rotinas, somada à distração causada pelos dispositivos, pode favorecer o consumo de alimentos ultraprocessados, especialmente bebidas açucaradas^(33)^. Soma-se o fato de que os hábitos alimentares dos pais podem influenciar esta ingestão, fazendo com que a criança consuma mais refrigerante ao comparar com outros alimentos, como frutas e vegetais^(13)^.

A associação entre maior tempo de tela e raça/cor parda evidencia desigualdades estruturais que impactam os hábitos digitais e alimentares das crianças. Estudos sugerem que crianças negras e pardas têm maior probabilidade de exposição a fatores de risco modificáveis, como tempo de tela prolongado e alimentação inadequada, refletindo a vulnerabilidade social e menor acesso a ambientes protetores^(34,35)^. A predominância de crianças declaradas pardas neste estudo ressalta a importância de considerar o recorte racial nas análises sobre práticas parentais, tempo de tela e comportamentos alimentares na primeira infância.

O período em que a criança frequenta a escola influencia diretamente seu tempo de exposição à televisão e ao *smartphone*. Observou-se que aquelas matriculadas em regime integral nos CEIs apresentaram menor uso de telas, reforçando o papel protetor da rotina escolar, já descrito em outros estudos^(36,37)^. Outro estudo acrescenta comparações entre diferentes idades, mostrando que crianças mais novas acumulam mais horas semanais de tela do que as mais velhas, sugerindo que a entrada e permanência em CEIs contribuem para reduzir gradualmente essa exposição^(38)^.

A elevada proporção de famílias beneficiárias de programas de transferência de renda evidencia a influência da vulnerabilidade socioeconômica na exposição infantil às telas. Embora crianças de famílias com maior poder aquisitivo tenham mais acesso a dispositivos, pesquisas indicam que, em contextos de vulnerabilidade, o uso das telas é ainda mais frequente^(39)^. Nesses contextos, as telas são frequentemente utilizadas como principal forma de lazer, devido à escassez de alternativas, o que pode agravar hábitos alimentares inadequados, conforme demonstrado por estudos nacionais e internacionais^(40,41)^.

Outro achado relevante foi a elevada proporção de mães com até oito anos de estudo, refletindo um cenário de baixa escolaridade materna. Esse fator, associado ao contexto socioeconômico desfavorável, pode influenciar tanto a exposição infantil às telas quanto os padrões alimentares, conforme já descrito em estudos nacionais e internacionais que relacionam menor escolaridade dos pais a práticas menos protetivas de saúde^(18,34,35)^.

A relação entre tempo de tela e imposição de limites também foi explorada. A maioria dos tutores relatou impor restrições ao uso de dispositivos, com maior taxa entre as crianças que permanecem até duas horas por dia em frente às telas. No entanto, não foi identificada associação estatisticamente significativa entre o estabelecimento desses limites e a redução do tempo de exposição, sugerindo que, mesmo diante da intenção declarada de controle, muitos cuidadores não conseguem efetivamente moderar o uso de telas pelas crianças. Esse resultado aponta para uma possível lacuna entre a prática parental e o comportamento real das crianças, achado que também foi observado em estudo canadense, no qual comparou-se a monitorização e limitação das telas durante a semana com o tempo real de exposição aos dispositivos, sendo a limitação imposta pelos pais não refletida diretamente em menor tempo de uso dos dispositivos^(42)^.

Diante disso, o consumo de doces diante das telas, mesmo com limites estabelecidos pelos tutores, é um achado preocupante. A ingestão de doces associada ao excesso de telas pode contribuir para padrões alimentares desregulados e maior vulnerabilidade a problemas de saúde ao longo da vida^(43)^. Viola *et al*.^(18)^ encontraram uma correlação entre o aumento do tempo de tela e a maior ingestão de alimentos ultraprocessados, como doces e bebidas açucaradas, sugerindo que o tempo prolongado em dispositivos pode atuar como um fator facilitador para a adoção de hábitos alimentares prejudiciais. Similarmente, Mumena *et al*.^(44)^ destacam que o uso excessivo de telas, especialmente nos períodos de lazer, está associado a um aumento do consumo de alimentos ricos em açúcar, corroborando a ideia de que o tempo de tela excessivo não apenas afeta o comportamento alimentar, mas também o desenvolvimento de hábitos alimentares de longo prazo.

Nesse contexto, o uso de telas e o consumo de guloseimas se mostram como práticas associadas a riscos cada vez mais incorporada às famílias, o que prejudica não apenas a criança em seu aspecto físico, mas também as características comportamentais, emocionais e sociais desenvolvidas ao longo da infância.

### Limitações do estudo

Por se tratar de delineamento transversal, o estudo não permite estabelecer relações causais entre tempo de tela, variáveis sociodemográficas e consumo de guloseimas. As associações observadas devem, portanto, ser interpretadas como correlacionais. Recomenda-se a condução de estudos longitudinais para melhor compreender os efeitos do tempo de exposição às telas sobre os padrões alimentares ao longo do desenvolvimento infantil. Além disso, a amostra foi composta por crianças matriculadas em CEIs da rede pública de um município nordestino, o que pode limitar a generalização dos achados para outras populações com diferentes perfis socioeconômicos e culturais. Além disso, o tempo de tela foi estimado por autorrelato dos responsáveis, o que pode ter introduzido viés de memória ou desejabilidade social. Por fim, não foram avaliados aspectos qualitativos do conteúdo nem o contexto de uso das telas, elementos relevantes para interpretar seus impactos no desenvolvimento.

### Contribuições para a área da enfermagem, saúde ou políticas públicas

Os achados deste estudo oferecem subsídios relevantes para a atuação de enfermeiros na Atenção Primária à Saúde e em unidades de educação infantil, com o objetivo de planejar ações preventivas voltadas à saúde digital e alimentar de crianças em idade pré-escolar. Nesse sentido, os enfermeiros podem incorporar, na rotina de cuidado, a avaliação do tempo de tela e dos hábitos alimentares durante consultas de puericultura, utilizando instrumentos breves de triagem e criando protocolos para identificar situações de risco. A atuação do enfermeiro pode incluir o desenvolvimento de oficinas educativas com pais e cuidadores, rodas de conversa, controle adequado da exposição às telas, além da elaboração de materiais educativos sobre alimentação saudável na primeira infância. Os enfermeiros também podem atuar de forma intersetorial, integrando ações de vigilância nutricional e orientação parental, fortalecendo políticas públicas voltadas à saúde integral da criança.

## CONCLUSÕES

O estudo evidenciou que as crianças pré-escolares que excedem duas horas diárias de tela apresentaram maior ingestão de refrigerantes e doces, sobretudo ao comer em frente às telas, com maior ocorrência nos fins de semana, sugerindo menor controle parental. Crianças em creches em tempo integral tiveram menor tempo de tela, indicando o papel protetor de ambientes e rotinas estruturadas.

Fatores estruturais, como baixa escolaridade materna, *status* socioeconômico desfavorável e autodeclaração racial parda, emergiram como determinantes críticos para a maior exposição às telas e padrões alimentares inadequados. Tais resultados reforçam a necessidade de intervenções intersetoriais, com ênfase na atuação da enfermagem no contexto da Atenção Primária à Saúde, voltadas à promoção de hábitos saudáveis de uso de telas e limitação de sua exposição durante as refeições, visando reduzir o consumo de guloseimas por pré-escolares.

## Data Availability

Os dados de pesquisa só estão disponíveis mediante solicitação.
